# The Cardenolide Glycoside Acovenoside A Interferes with Epidermal Growth Factor Receptor Trafficking in Non-Small Cell Lung Cancer Cells

**DOI:** 10.3389/fphar.2021.611657

**Published:** 2021-05-05

**Authors:** Susanne Hafner, Michael Schmiech, Sophia Johanna Lang

**Affiliations:** Institute of Pharmacology of Natural Products and Clinical Pharmacology, Ulm University, Ulm, Germany

**Keywords:** cardenolide glycoside, apoptosis, Na^+^/K^+^-ATPase, non-small cell lung cancer, EGFR, ubiquitination, endosomal arrest

## Abstract

Cardenolide glycosides are natural compounds known to inhibit the ion pumping function of the Na^+^/K^+^-ATPase in cellular systems. Interestingly, various cancer cell types are highly susceptible to cardenolide glycosides. Herein, we explore the cardenolide glycoside Acovenoside A (AcoA) with respect to its influences on human A549 non-small cell lung cancer (NSCLC) cells. We found that exposure to AcoA, digoxin and ouabain increases intracellular sodium and ATP levels indicating that the ion pumping function of the transmembrane Na^+^/K^+^-ATPase is effectively inhibited. Like digoxin and ouabain, AcoA inhibits transcription factor NF-κB activation and induces apoptotic cell death in NSCLC cells. This was confirmed by a preclinical *in vivo* model in which AcoA treatment of NSCLC xenografts grown on chick chorioallantoic membranes inhibited the expression of proliferation antigen Ki-67 and induced apoptotic DNA strand breaks. We aimed to elucidate the underlying mechanisms. The Na^+^/K^+^-ATPase transmembrane complex contains Src kinase and epidermal growth factor receptor (EGFR). Indeed, we found that AcoA activates Src kinase in A549 cells, but not in a cell-free assay using recombinant Src kinase. Src kinase is a downstream target of EGFR, and correlation analysis using the NCI60 database pointed to a role of EGFR in cardenolide glycoside-induced cancer cell death. Accordingly, NSCLC cells expressing hyperphosphorylated EGFR^mut^ exhibited resistance to AcoA. To investigate the interaction between cardenolide glycosides and EGFR in detail, we performed immunoblotting studies: Whereas ligand binding and EGFR phosphorylation were not significantly affected, ubiquitinated EGFR accumulated after prolonged incubation with AcoA. To visualize EGFR trafficking we used A549 cells transfected with a fluorescent biosensor which binds to activated EGFR. Pretreatment with AcoA and digoxin induced accumulation of EGFR in endosomal compartments thus inhibiting EGF-induced EGFR degradation comparable to the Na^+^ ionophore monensin, a known inducer of EGFR endosomal arrest. Intracellular Na^+^ concentrations regulate EGFR trafficking and signaling. Na^+^ homeostasis is maintained by the Na^+^/K^+^-ATPase, which might account for its close interaction with the EGFR. Cardenolide glycosides inhibit the ATP-dependent Na^+^/K^+^ exchange through the Na^+^/K^+^-ATPase resulting in higher intracellular Na^+^ levels. Our data provide first evidence that this impedes efficient EGFR trafficking at the endosomal compartment.

## Introduction

The term cardenolide glycoside is used for a diverse group of naturally derived substances composed of a steroid skeleton linked to a sugar moiety at the C3 position and a lactone substituent at the C17 position (Diederich et al., 2017). Therapeutic administration of certain cardenolide glycosides to improve cardiac ejection performance has a long-standing history, and despite considerable risks of serious adverse effects and intoxication, cardenolide glycosides are still part of international guidelines for the treatment of heart failure and atrial fibrillation (Ponikowski et al., 2016; Ziff and Kotecha, 2016).

Cardenolide glycosides target the α-subunit of Na^+^/K^+^-ATPases located on cellular membranes. By inhibition of the ATPase, they affect ionic fluxes through cellular transporters increasing intracellular sodium and calcium concentrations. The ATP-consuming ion pumping function of the Na^+^/K^+^-ATPase transporting 3 Na^+^ out of and 2 K^+^ into the cell was early understood and utilized. In the past two decades, the Na^+^/K^+^-ATPase was re-discovered as a cellular signaling receptor. The molecular functions ascribed to “non-pumping” Na^+^/K^+^-ATPase reach far beyond ionic gradients and comprise regulation of protein kinase cascades, transcription factors, membrane transporters, and receptors (Liang et al., 2007). Thus, by interfering with Na^+^/K^+^-ATPase-related processes, cardenolide glycosides in the nanomolar range are affecting essential mechanisms of cell metabolism, e.g. transcription and translation, glycolysis and immune responses, and ultimately can direct cellular fate towards either proliferation or death (Sepp et al., 2014; Diederich et al., 2017; Orlov et al., 2017).

Since the first clinical data of breast cancer patients in the late 1970ies suggested an enhanced susceptibility of cancer cells to cardenolide glycosides (Stenkvist et al., 1979), experimental evidence has been generated demonstrating antiproliferative efficacy of these compounds in malignant, but not in healthy cells (Diederich et al., 2017). For this reason, research on cardenolide glycosides has shifted its focus from cardiology to oncology. Targeting Na^+^/K^+^-ATPase by cardenolide glycosides has been suggested for the prevention and treatment of various proliferative diseases (Chen et al., 2006; Mijatovic et al., 2007b; Newman et al., 2008).

As clinical samples from non-small cell lung cancer (NSCLC) often show overexpression of the α1-subunit, targeting Na^+^/K^+^-ATPase has been suggested in particular to treat lung cancer (Mijatovic et al., 2007a; Schneider et al., 2017). Accordingly, many cardenolide glycoside-related studies use A549 NSCLC cells as a tumor cell model (Schneider et al., 2018; Triana-Martinez et al., 2019).

Concerning cardenolide glycosides’ mode of action, there is evidence of cardenolide glycoside-induced activation of Src kinase, activation of the Ras-Raf-MAP kinase pathway, inhibition of transcription factor NF-κB, and production of reactive oxygen species (ROS) with subsequent mitochondrial damage (Newman et al., 2008; Prassas and Diamandis, 2008). However, despite the considerable number of mechanisms demonstrably involved, it is still unclear which one is critical to trigger apoptotic or autophagic cancer cell death and, which intracellular target might determine the differential actions of cardenolide glycosides in cancer vs. normal cells. To date, only a small number of cardenolide glycosides has been fully characterized. However, since the Na^+^/K^+^-ATPase was recognized as the “Achilles heel” of multidrug resistant tumor cells (Mijatovic et al., 2009) there is a growing scientific and clinical interest in new candidates providing higher tolerability and selectivity than classical digoxin or ouabain/strophantin.

In previous work, we have isolated the cardenolide glycoside acovenoside A (AcoA) from the pericarps of *Acokanthera oppositifolia*, performed substance characterization and toxicological studies in rodents, and found anti-inflammatory efficacy of AcoA in a murine model of doxorubicin-induced cardiotoxicity (Ezzat et al., 2016). Besides, we have shown that AcoA is non-toxic to peripheral blood mononuclear cells, whereas in NSCLC cells, AcoA-induced cell death was accompanied by the production of ROS (El Gaafary et al., 2017). Nonetheless, ROS scavenging did not rescue NSCLC cells indicating that ROS production is not a major contributor to AcoA-induced cell death. Thus, the actual mechanism of AcoA toxicity in cancer cells has not been elucidated, yet.

In the present work, we explore pharmacodynamic features of AcoA and draw comparisons with digoxin and ouabain as the most common members of the cardenolide glycoside family. We further shed light on the underlying mechanisms of AcoA-induced toxicity in NSCLC cells and investigate how AcoA affects the trafficking of the epidermal growth factor receptor (EGFR).

## Materials and Methods

### Reagents and Equipment

The cardenolide glycosides AcoA and ouabain were purchased from Sigma (St. Louis, MO), digoxin from Tocris Bioscience (Bristol, United Kingdom). Doxorubicin hydrochloride and the EGFR tyrosine kinase inhibitor erlotinib were from Cayman Chemical (Ann Arbor, MI). The monoclonal EGFR antibody cetuximab (Erbitux^®^) was purchased from Merck (Merck, Darmstadt, Germany). The Src kinase inhibitor PP2 **(**4-amino-3-(4-chlorophenyl)-1-(t-butyl)-1H-pyrazolo[3,4-d]pyrimidine) was bought from Enzo (Enzo Life Sciences, Lausen, Switzerland), staurosporine was from Sigma (St. Louis, MO). Human recombinant TNF-α and EGF were from PeproTech (Hamburg, Germany). Stock solutions of AcoA and other substances were prepared in DMSO and further diluted with medium supplemented with 1% heat-inactivated fetal calf serum.

Absorbance, fluorescence, and luminescence were measured with an Infinite M1000 PRO plate reader (Tecan Group, Maennedorf, Switzerland). For flow cytometric measurements, a FACSVerse flow cytometer (Becton Dickinson, Heidelberg, Germany) was used.

### Cell Lines

Basal human A549 NSCLC cells were purchased from ATCC (Rockville, MD) and cultured in F-12K medium.

A549 Red-FLuc cells stably transfected with the firefly luciferase gene from *Luciola Italica* (PerkinElmer, Waltham, MA) were cultured in RPMI-1640 supplemented with 2 mM L-glutamine and 2 μg/ml puromycin.

NF-κB reporter cells for the study of the NF-κB pathway (A549-Dual™ cells, InvivoGen, San Diego, CA) express a secreted embryonic alkaline phosphatase (SEAP) reporter gene under the control of the IFN-β minimal promoter fused to five NF-κB binding sites. NF-κB reporter cells were grown in DMEM supplemented with 2 mM L-glutamine, 4.5 g/L glucose, 100 μg/ml normocin and the selection antibiotics blasticidin (10 μg/ml) and Zeocin (100 μg/ml).

To visualize EGFR trafficking, we used A549 EGFR biosensor cells from Sigma (Malkov et al., 2011; Antczak and Djaballah, 2016). These cells were stably transfected with a biosensor consisting of a fusion protein of two Src-homology 2 (SH2) domains of growth factor receptor bound protein 2 (Grb2) and a green fluorescent protein tag. Stimulation of the receptor by EGF and subsequent tyrosine phosphorylation allows binding of the biosensor to phosphotyrosine residues of the receptor kinase domain. This is followed by internalization of EGFR-bound GFP-Grb2-SH2 with intracellular granule formation. The fluorescent tag facilitates microscopical imaging and quantification. The biosensor only responds to specific EGFR ligands and the assay has been validated against a library of almost 7000 substances including EGFR activators and inhibitors (Antczak et al., 2012). The EGFR biosensor cells were grown in RPMI-1640 supplemented with 2 mM L-glutamine and 1 μg/ml puromycin.

NSCLC HCC-827 cells exhibiting an acquired mutation in the tyrosine kinase domain of the EGFR (E746-A750 deletion, EGFR^mut^) and NSCLC CAL-12T EGFR^wt^ (both from DSMZ, Braunschweig, Germany) were cultured in RPMI-1640 or DMEM, respectively.

### Measurement of Intracellular Na^+^ Levels

The fluorescent Na^+^ indicator ANG-2 (Asante NaTRIUM Green, Teflabs Inc., Austin, TX) was used to quantify intracellular Na^+^ changes. For loading, stimulation, and measurement, Hank’s Balanced Salt Solution (HBSS) containing 1% BSA was used. A549 cells seeded in 96-well plates the day before the experiment (5,000 cells/well) were incubated with loading buffer containing ANG-2 acetoxymethyl pre-diluted (1:1) with pluronic F-127 (20% in DMSO) at 37°C for 1 h. After washing and addition of compounds or vehicle (loading buffer containing 0.5% DMSO), fluorescence kinetics were recorded (excitation 532 nm/emission 548 nm) at constantly controlled temperature of 37°C using a fluorescence plate reader. Na^+^ kinetics were recorded every 2 min for 60 min and after 120 min. To correct for background fluorescence, unloaded cells treated in the same way as the loaded samples were measured in parallel and the fluorescence intensity subtracted from the mean fluorescence intensity of the respective loaded samples at each time point. Visualization of Na^+^ kinetics: To correct for signal changes due to bleaching and vehicle-dependent effects, the delta of the mean fluorescence intensity of loaded cells treated with vehicle only was subtracted at every time point. Na^+^ levels after 120 min were expressed as fold-change related to the baseline fluorescence of each sample after subtraction of background (unloaded cells).

### Analysis of Cellular NF-κB Transcription Factor

Commercially available NF-κB reporter cells (A549-Dual™ cells) allow for the analysis of NF-κB response by assessing the activity of a secreted embryonic alkaline phosphatase (SEAP) in cell supernatants after addition of an SEAP detection reagent (QUANTI-Blue™) by absorbance measurement (OD 630 nm). 25,000 cells/well seeded the day before the experiment were treated with the respective compounds. 30 min later, pathway activator TNF-α (100 ng/ml) was added. After the indicated periods, supernatants were collected and analyzed for the reporter expression. To exclude non-specific effects of the tested compounds, cell viability was determined by XTT assay.

### Cell Viability, Metabolic Activity, and Apoptosis Analysis

To measure cell proliferation and viability the XTT assay (Roche Diagnostics, Filderstadt, Germany) based on the colorimetric quantification of formazan dye metabolized by mitochondrial dehydrogenases was used. Cellular ATP levels were determined in luciferase-expressing A549 cells 10 min after addition of D-luciferin (Biomol, Hamburg, Germany). Apoptotic cells were quantified using flow cytometric analysis of annexin V-FITC/propidium iodide double-stained cells. The data were analyzed using FlowJo software. Cell-cycle analysis was performed according to the method of Nicoletti et al. (Nicoletti et al., 1991) to evaluate the percentage of subdiploidal G1 cells (subG1) carrying fragmented DNA indicative of apoptosis.

### Lung Cancer Xenografts on the Chorioallantoic Membrane of Fertilized Chick Eggs

To investigate tumor response to AcoA in an *in vivo* microenvironment, we used the chorioallantoic membrane (CAM) of fertilized chick eggs. Although the CAM model does not replace further animal studies in rodents, it provides valuable preclinical information. In particular, it enables the formation of solid tumors with vascularization and allows the rapid and direct evaluation of tumor size and angiogenesis by a reproducible and efficient method (Saw et al., 2008; Ribatti, 2014). *In vivo* CAM experiments were performed as previously described (Wu et al., 2015). Briefly, 1 x 10^6^ A549 NSCLC cells were xenotransplanted in medium/matrigel (1:1, v/v) onto the chick CAM 7 days after fertilization. Starting the next day, the xenografts were topically treated for 3 consecutive days with 20 µL of each sample. After histological preparation, serial sections (5 µm) were stained for the proliferation antigen Ki-67 (Dako Cytomation, Glostrup, Denmark) using a commercial staining kit (Histostain-Plus, Invitrogen) according to the manufacturer’s instructions. To avoid non-specific background, a ready-to-use blocking solution consisting of 10% goat non-immune serum was applied prior to incubation with the primary antibody (monoclonal mouse, anti-human Ki-67 antigen, M7240, Dako Cytomation, Glostrup, Denmark) at a dilution of 1:80 in antibody diluent for 1 h at 37°C. This was followed by application of biotinylated secondary antibody (30 min at RT), and horseradish peroxidase conjugated streptavidin (15 min at RT). Upon application of the chromogen aminoethyl carbazol (AEC Solution, Lifetechnologies), the peroxidase catalyzes the substrate (hydrogen peroxide) and converts the chromogen to red deposit, which visualizes the location of Ki-67. The slides were counterstained by hematoxylin, shortly immersed in 37 mM NH_4_OH in water, and mounted using Aquatex aqueous mounting media.

For the detection of apoptotic cells in NSCLC xenografts, DNA strand breaks were visualized by the terminal deoxynucleotidyl transferase (TdT)-mediated dUTP biotin nick-end labeling (TUNEL) method using an *In situ* Cell Death Detection Kit POD from Roche. For protease treatment, the tumor sections were incubated with proteinase K (30 μg/ml) for 10 min at 37°C. The TUNEL reaction mix was prepared consisting of equal volumes of enzyme solution (with recombinant terminal deoxynucleotidyl transferase) and label solution (a nucleotide mixture in reaction buffer) and applied for 1 h. A slide incubated with pure label solution instead of the terminal deoxynucleotidyl transferase enzyme-containing reaction mix served as a negative control. After washing, the signal was converted to brown deposit using Converter-POD (anti-fluorescein antibody, Fab fragment from sheep, conjugated with horseradish peroxidase) and DAB substrate (Roche). Counterstaining and mounting was performed as described above.

To assess the percentages of Ki-67 and TdT positive cells, the numbers of positive and negative cells were manually counted by a technical assistant blinded to the treatment protocol. Per tumor, at least 2 fields of view were assessed.

All *in vivo* study protocols complied with the National and European Union guidelines for animal experiments (directive 2010/63/EU).

### Proteome Profiling and EGFR Phosphorylation Arrays

To detect AcoA-mediated changes of cellular signaling pathways, various arrays were used according to the manufacturer’s instructions: human phospho-kinase (catalogue number ARY003C), phospho-RTK (ARY001B), ubiquitin (ARY027), cell stress array (ARY018); all by R&D Systems, Wiesbaden-Nordenstadt, Germany. To evaluate EGFR phosphorylation at distinct phosphorylation sites we used EGFR phosphorylation antibody arrays (Abcam, Cambridge, United Kingdom). Cell lysates were incubated with capture and control antibodies spotted on nitrocellulose membranes. The membranes were exposed to autoradiography film or an Amersham Imager 600RGB (GE Healthcare, Buckinghamshire, United Kingdom). Pixel density was evaluated using ImageJ analysis software. After background subtraction, the signal was compared to the corresponding signal of the control array. Additionally, phospho-EGFR or ErbB2 signals were normalized to the pan EGFR or ErbB2 signals.

### Microscopic Monitoring of EGFR Trafficking

For microscopical evaluation of EGFR trafficking, we used A549 EGFR biosensor cells with an SH2 domain of the adaptor protein Grb2 fused to GFP. Upon stimulation with EGF, EGFR is internalized by endocytosis forming fluorescent granules. A549 biosensor cells were seeded in ibidi 8-well µ-slides (10,000 cells/well) and exposed to AcoA or control compounds. After 1 h, EGF (100 ng/ml) was added. Cell nuclei were counterstained with DAPI-containing mounting medium (Vectashield, Vector Laboratories, La Jolla, CA) and cells were viewed under a Nikon TS Ti-E fluorescence microscope using NIS Elements Advanced Research Microscope Imaging Software (Nikon Corporation, Tokyo, Japan) for imaging and image processing. The number of granules was normalized to the number of DAPI-stained nuclei in the respective area.

### Quantification of Total EGFR and *p*-EGFR^Y1173^

To confirm our findings, we quantified EGFR in A549 cells using commercial EGFR ELISA assays (Phospho-EGFR (Y1173) + Total In-Cell ELISA Kit, Abcam, Cambridge, UK). The assays were performed according to the manufacturer’s instructions. Briefly, cells were seeded in 96-well plates (20,000 cells/well) and incubated overnight. Upon substance application and incubation for the desired time, the cells were washed with PBS and fixed. After blocking, primary antibody recognizing EGFR only when phosphorylated at the Tyr1173 phosphorylation site (*p*-EGFR^Y1173^) or primary antibody against EGFR protein regardless of its phosphorylation state (EGFR^total^) was applied for overnight at 4°C on a shaking platform. After washing, horseradish peroxidase conjugated IgG (secondary antibody) was added for 1 h at RT. After another washing step, a TMB substrate solution was added to develop blue color in proportion to the amount of *p*-EGFR or EGFR, respectively. Addition of stop solution turned the color to yellow, and the absorbance was measured at 450 nm using a plate reader. Afterwards, the cells were washed again, and stained with crystal violet to normalize the EGFR signal to the number of analyzed cells. The *p*-EGFR signal was normalized to the EGFR^total^ signal.

### Measurement of Src Kinase Activity and Phosphorylation

CycLex® inhibitor screening kits were used to analyze whether AcoA affects the activity of recombinant Src kinase directly. CycLex® Src kinase assay/inhibitor screening kits and the recombinant catalytic domain of c-Src were from MBL (MBL International, Woburn, MA).

Substances were diluted to 100 nM in freshly prepared kinase reaction buffer containing Mg^2+^, Mn^2+^ and ATP (10 µM) and preincubated with Src kinase at RT for 10 min. The mixtures were added to substrate-coated wells for 30 min at 30°C. The tyrosine kinase inhibitor staurosporine served as a positive control. Enzyme activities were quantified spectrophotometrically at 450/540 nm using a Tecan Infinite M1000 Pro microplate reader.

To measure Src kinase phosphorlyation, we used an ELISA Kit (PathScan Phospho-Src (Tyr416) Sandwich ELISA Kit, Cell Signaling Technology). A549 cells seeded in Petri dishes were treated with compounds for the desired time. Then, media were removed, the plates were put on ice and rinsed with ice-cold PBS. Cells were lysed in cell lysis buffer (containing 20 mM Tris-HCl (pH 7.5), 150 mM NaCl, 1 mM Na_2_EDTA, 1 mM EGTA, 1% Triton, 2.5 mM sodium pyrophosphate, 1 mM beta-glycerophosphate, 1 mM Na_3_VO_4_, 1 μg/ml leupeptin, and 1 mM PMSF) for 5 min, scraped and transferred to a tube. After sonication and centrifugation at 14,000 g for 10 min, the supernatants were collected and protein concentration was measured by BCA assay (Micro BCA Protein Assay kit, ThermoFisher Scientific). The samples were diluted to a protein concentration of 0.3 mg/ml in sample diluent and added to microwells coated with a phospho-Src rabbit antibody. After incubation with cell lysates overnight at 4°C, a Src mouse detection antibody was applied. Horseradish peroxidase-linked anti-mouse antibody served to recognize the bound detection antibody. TMB substrate solution was added for color development and upon addition of stop solution, absorbance was measured by a plate reader at 450 nm.

### Statistical Analysis

Statistical analysis was performed using Statistica software (StatSoft, Tulsa, OK) or SigmaPlot software 12.5 (Systat Software GmbH, Erkrath, Germany). IC_50_ values of 50% growth inhibition were assessed by non-linear regression. Multi-group comparisons were performed by Kruskal-Wallis one-way analysis of variance (ANOVA) with Newman-Keuls posthoc test, alpha = 0.05. *p* values are expressed as *p < 0.05, **p < 0.01 and ***p < 0.001. Data are presented as means ± SEM (standard error of the mean) or SD (standard deviation) of N independent experiments or samples (*N* ≥ 3). Further details on each data set can be found in the respective figure legend.

## Results

**FIGURE 1:**
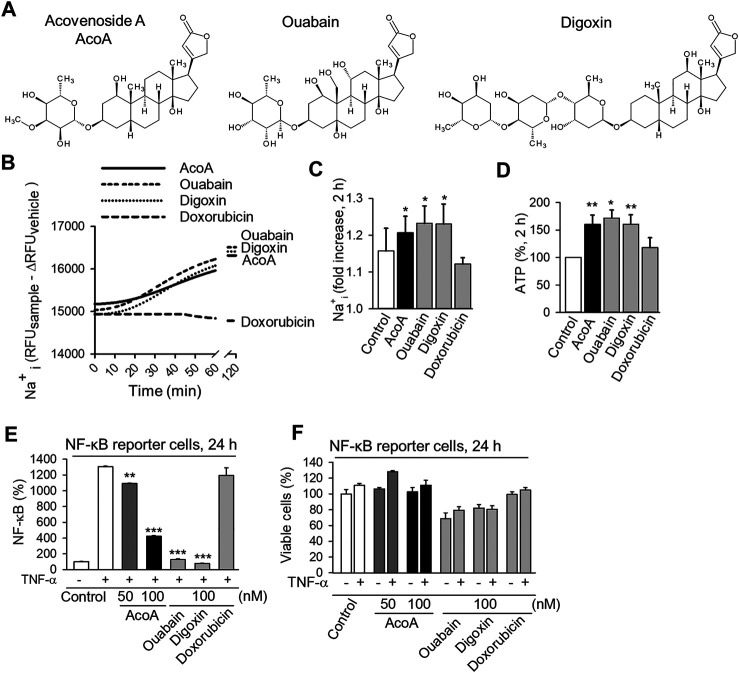
AcoA shows typical features of the cardenolide glycoside family by increasing intracellular Na^+^ concentration and inhibiting NF-κB activation in A549 lung cancer cells. **(A)** Chemical structures of acovenoside A (AcoA), ouabain, and digoxin were designed using Bio Draw software by Dassault Systèmes. **(B)** Intracellular Na^+^ changes after the addition of 100 nM AcoA, ouabain, digoxin, or solvent (control) were quantified using ANG-2 fluorescent Na^+^ indicator. The MFI values were corrected for background via subtraction of the MFI of unloaded cells treated in the same way as loaded cells. To correct for bleaching and vehicle-dependent effects, the delta MFI of vehicle-treated cells (MFI of vehicle-treated cells at time x minus MFI of vehicle-treated cells at time 0) was subtracted at every timepoint. Kinetics were recorded continuously every 2 min for 60 min using a fluorescence plate reader. **(C)** Intracellular sodium increase 2 h after substance application (all 100 nM). After subtraction of the fluorescence of unloaded samples (as in B) the MFI values were normalized to the baseline fluorescence of the respective sample. Data are mean ± SEM of *N* = 3–4. **(D)** ATP levels increase in luciferase-expressing A549 cells 2 h after substance application (all 100 nM). ATP levels were analyzed after addition of luciferin. Data are mean ± SEM of *N* = 4. **(E)** AcoA, ouabain and digoxin inhibit TNF-α induced NF-κB activation. NF-κB reporter cells were treated with AcoA (50 nM or 100 nM), ouabain, and digoxin (both 100 nM) and stimulated with TNF-α (100 ng/ml) for 24 h. Data are mean ± SD of triplicates, **p < 0.01; ***p < 0.001 vs. the positive control group treated with TNF-α. **(F)** The viability of NF-κB reporter cells after 24 h under the chosen experimental conditions was only slightly compromised. This indicates that the effects on NF- κB activation are not only due to cytotoxicity (mean ± SD of triplicates).

We aimed to compare AcoA to other cardenolide glycosides and chose digoxin and ouabain as the most common members of the cardenolide glycoside family. The chemical structures are presented in Figure 1A. To predict physicochemical and pharmacokinetic properties, the validated webtool SwissADME databank was utilized (Daina et al., 2017), http://www.swissadme.ch. The analysis is depicted in Table 1 and revealed hydrophilic properties for ouabain, whereas AcoA and digoxin are more lipophilic. All three cardenolide glycosides are not likely to pass the blood brain barrier. However, in contrast to digoxin and oubain, AcoA is expected to be highly absorbed through the gastrointestinal mucosa.

**TABLE 1:** Predicted physicochemical and pharmacokinetic properties of the investigated cardenolide glycosides and doxorubicin according to SwissADME*.

Compound	Mw (g/mol)	Lipophilicity (LogP)**	Water Solubility	GI absorption	BBB permeant	P-gp substrate
AcoA	550.68	2.01	Soluble	High	No	Yes
Digoxin	780.94	1.85	Moderately soluble	Low	No	Yes
Ouabain	584.65	−0.55	Soluble	Low	No	No
Doxorubicin	543.52	0.44	Soluble	Low	No	Yes

Abbreviations: Mw, molecular weight; GI, gastrointestinal; BBB, blood brain barrier; P-gp, P-glycoprotein.

*By Swiss Institute of Bioinformatics, http://www.swissadme.ch, accessed September 9, 2020.

**Consensus LogP: average of 5 predictions.

### AcoA Increases Intracellular Na^+^ Concentration and ATP Levels

We examined AcoA from a pharmacodynamic perspective. The Na^+^/K^+^-ATPase is considered the main target of cardenolide glycosides and depends on ATP to pump sodium (Na^+^) out of the cell (Durlacher et al., 2015; Diederich et al., 2017). Inhibition of its ion pumping function results in Na^+^ being retained intracellularly consequently increasing intracellular Na^+^ concentrations. We investigated the effect of AcoA on the ion pumping function of the Na^+^/K^+^-ATPase by monitoring intracellular Na^+^ levels. Within 30 min of exposure of A549 cells to AcoA, ouabain, or digoxin, Na^+^ levels started to steadily increase, whereas doxorubicin, a chemotherapeutic without impact on Na^+^/K^+^-ATPase activity, did not affect cellular Na^+^ concentrations (Figures 1B,C). The observed Na^+^ increase was accompanied by rising cellular ATP levels (Figure 1D).

### AcoA Inhibits TNF-α-Induced NF-κB Activation

In addition to inhibiting the Na^+^/K^+^-ATPase ion pumping function, cardenolide glycosides affect the activity of transcription factors and the inhibition of the NF-κB pathway has emerged as a hallmark of cardenolide glycoside pharmacodynamics in cancer cells (Diederich et al., 2017; Schneider et al., 2017). In particular, ouabain and digitoxin have been shown to reduce TNF-α-induced NF-κB activation (Miller et al., 2010). To examine if this applies for AcoA as well, we used a NF-κB reporter cell line expressing a reporter gene under the control of the IFN-β minimal promoter fused to five NF-κB binding sites. Upon incubation with AcoA, we observed a significant and concentration-dependent inhibition of TNF-α-stimulated NF-κB activation after 24 h (Figure 1E). Both, ouabain and digoxin, significantly reduced NF-κB as well and even to a greater extent than AcoA, whereas the conventional chemotherapeutic doxorubicin did not affect NF-κB. The assessment of cell viability at this time point ensured that the observed changes were not a mere by-product of cell death (Figure 1F). In contrast to unmodified A549 cells, viability of the NF-κB reporter cell line was only slightly reduced by cardenolide glycosides at this time point.

### AcoA Inhibits Proliferation and Induces Apoptosis in NSCLC Cells *in vitro* and *in vivo*

**FIGURE 2:**
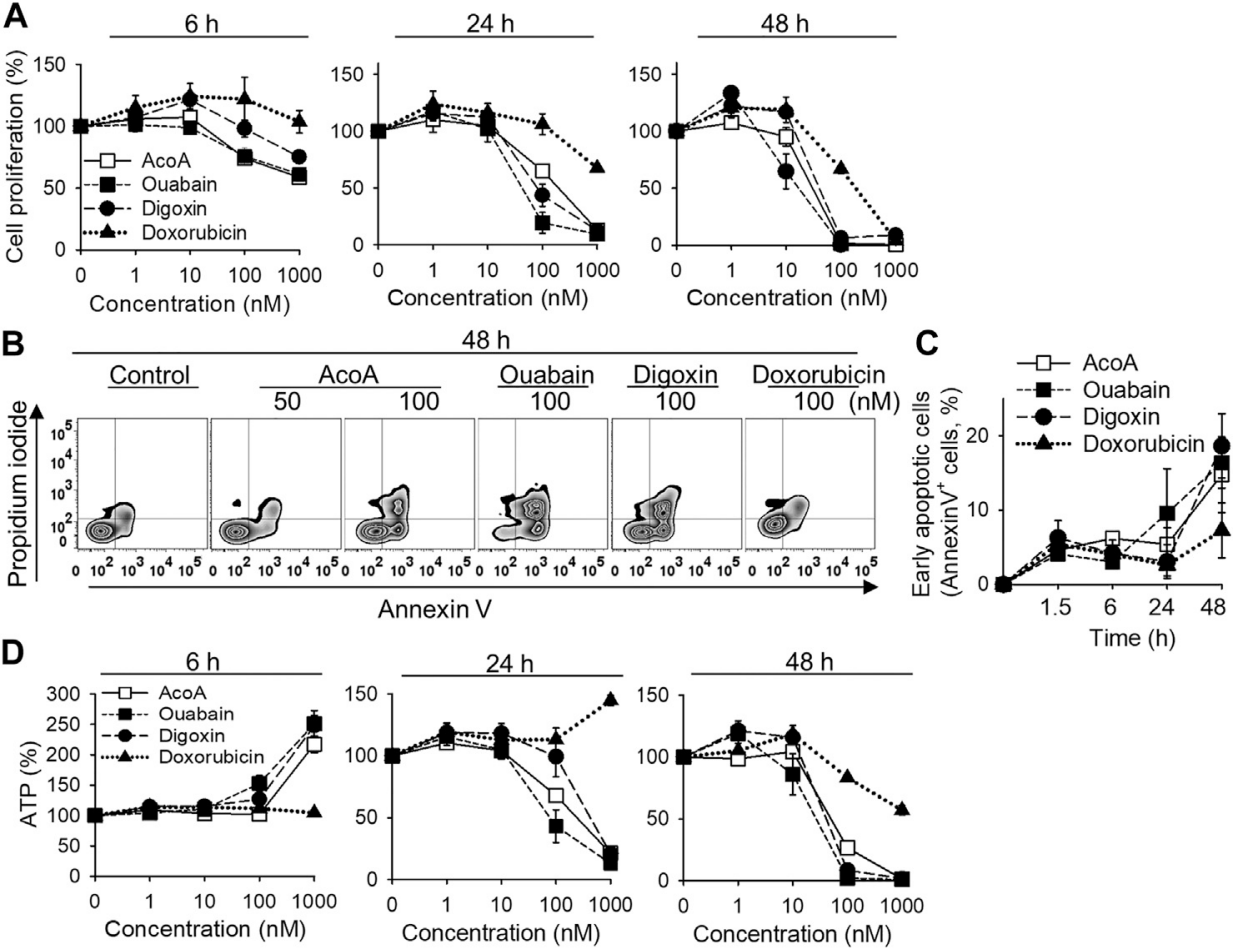
AcoA induces apoptosis in A549 lung carcinoma cells *in vitro*. **(A**) A549 lung cancer cells were treated with different concentrations of AcoA, ouabain, digoxin, or doxorubicin and cell viability was measured by XTT. **(B)** To detect apoptosis, A549 cells were stained with FITC-annexin V and propidium iodide and analyzed by flow cytometry. The graph shows representative flow cytometry histograms after 48 h incubation with the respective compounds. **(C)** Percentage of early apoptotic cells (annexin V^+^, PI^−^). **(D)** Effect of AcoA, ouabain, and digoxin on ATP levels in A549 cells. ATP in luciferase-expressing A549 cells was analyzed after addition of luciferin. All data are mean ± SEM of *N* = 3 independent experiments performed in triplicates.

We previously found that AcoA-induced toxicity in NSCLC cells is accompanied by caspase 3 and cell cycle arrest indicating apoptosis (El Gaafary et al., 2017). We now aimed to confirm and further explore the cytotoxic potential of AcoA in NSCLC cells in direct comparison to ouabain and digoxin (Figure 1A). Metabolic activity of NSCLC cells was reduced by AcoA, ouabain and digoxin in a time- and concentration-dependent manner (Figure 2A). After 48 h, AcoA, ouabain, and digoxin induced apoptotic changes in 14.8, 18.6, and 16.4% of the cell population, respectively (Figures 2B,C). Doxorubicin, a commonly used chemotherapeutic drug, was less efficient at equimolar concentrations. Interestingly, while mitochondrial activity after 6 h of exposure to cardenolide glycosides was reduced, the concurrently measured bioluminescence, which correlates to cellular ATP levels, increased by more than 100% (Figure 2D).

**FIGURE 3:**
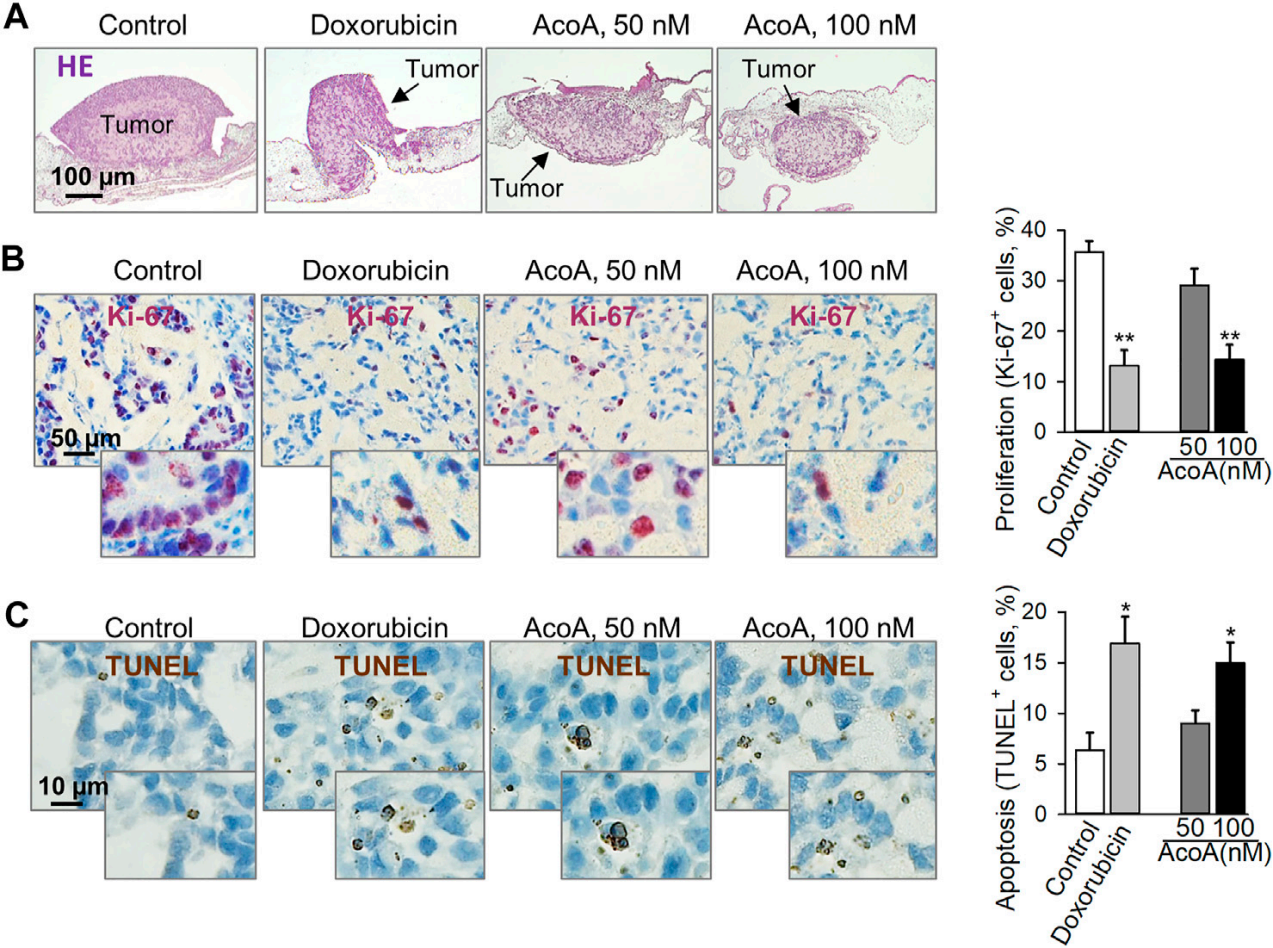
AcoA inhibits tumor growth and induces apoptosis in A549 lung cancer xenografts on the chorioallantoic membrane of fertilized chick eggs *in vivo*. 1 × 10^6^ A549 cells were xenotransplanted onto the chorioallantoic membrane of fertilized chick eggs 7 days after fertilization. Starting the next day, the tumors were treated with AcoA, doxorubicin, or solvent (control) for 3 days. **(A)** Hematoxylin and eosin staining, original magnification 50x. Representative pictures are shown. **(B)** Immunohistochemical analysis of tumor cell proliferation using nuclear Ki-67 antigen (proliferation marker, dark violet). Nuclei were counterstained with hematoxylin (blue). To assess the percentage of Ki-67 positive cells, the numbers of Ki-67-positive and negative cells were counted. Per tumor, at least 2 fields of view were assessed corresponding to 208 to 503 cells. Original magnification 200x, *N* = 5 eggs/group. **(C)** TUNEL staining (brown) to detect cells with fragmented DNA as apoptosis marker. In each tumor section, 226–434 cells were counted. Original magnification 400x, *N* = 5 eggs/group. Data are mean ± SEM, *p < 0.05, **p < 0.01 in comparison to the vehicle-treated control group.

Most preclinical *in vivo* studies are performed in rodents. When using rodents for cardenolide glycoside-related research one should bear in mind that Na^+^/K^+^-ATPases from mouse, rat, or hamster are more than 1000 times less sensitive to cardenolide glycosides than human cells (Newman et al., 2008; Calderon-Montano et al., 2014), and toxicity results in rodents might not be valid for other species. Accordingly, mouse-derived J774.1 cells showed resistance to AcoA and lacked typical effects on cellular NF-κB levels even at elevated concentrations up to 100 µM (Supplementary Figure S2). Here, we used the chorioallantoic membrane of fertilized chick eggs to establish solid lung cancer xenografts. AcoA inhibited the growth of A549 xenografts as reflected by significantly reduced tumor size (Figure 3A, Supplementary Figure S3). Immunohistochemical analysis revealed that AcoA treatment significantly inhibited the expression of the Ki-67 proliferation antigen (Figure 3B) and induced DNA strand breaks indicating apoptosis in lung cancer xenografts (Figure 3C). Doxorubicin served as a control, and in comparison to AcoA, doxorubicin had similar anticancer efficacy and was even more efficient than in our *in vitro* studies (Figure 2). The latter is most likely due to a higher concentration of free doxorubicin because the compounds were solved in physiological saline solution instead of albumin-containing cell culture medium.

### AcoA Phosphorylates Src Kinase in A549 Cells, but Reduces the Activity of Recombinant Src Kinase

**FIGURE 4:**
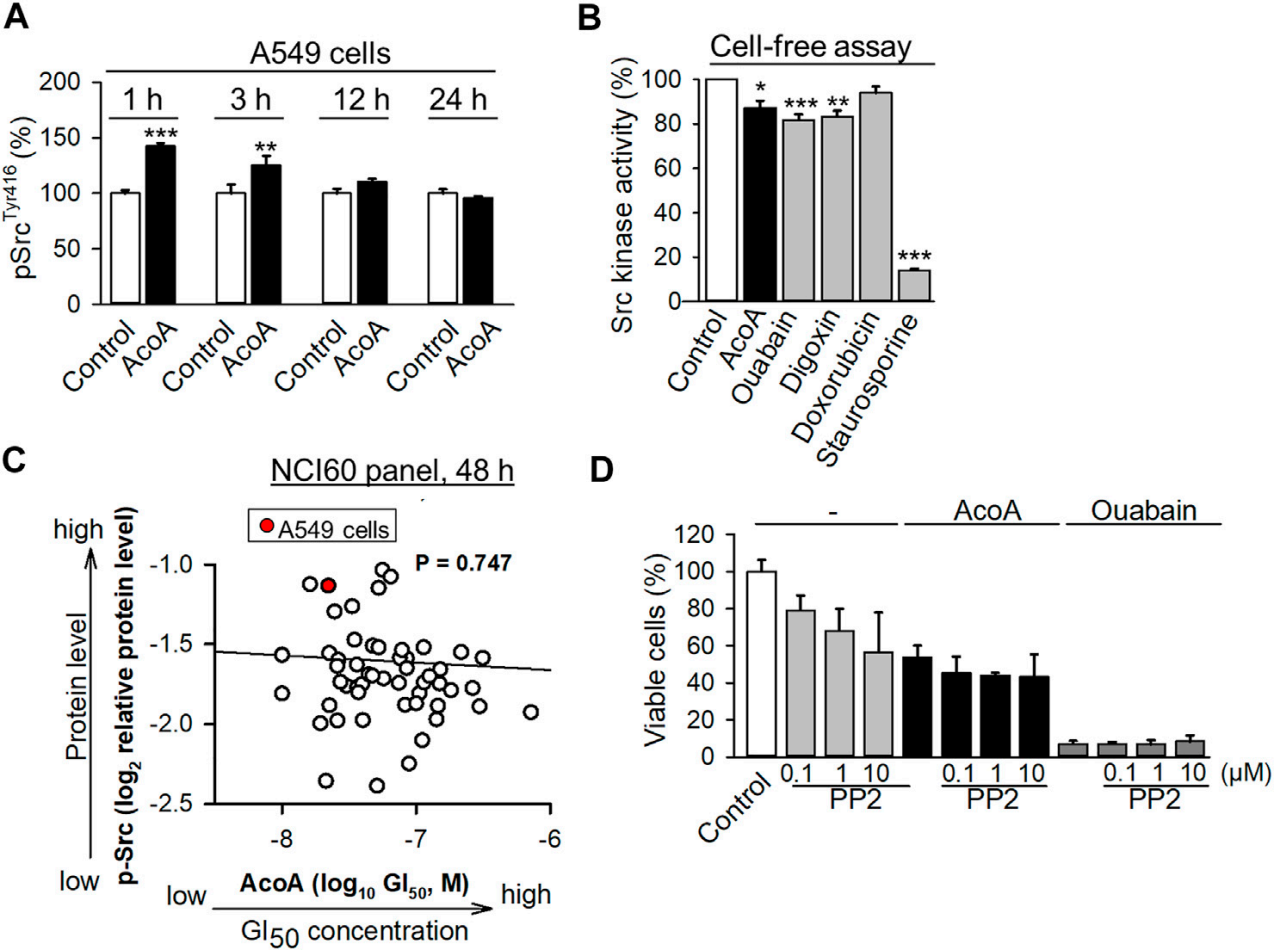
AcoA activates Src kinase in A549 cells, but reduces the activity of recombinant Src kinase in a cell-free assay. **(A)** A549 cells were treated with AcoA for 1 to 24 h, lysed and the cell lysates were analyzed for activation of the Tyr416 phosphorylation site of Src kinase using a commercial ELISA assay. Data are mean ± SEM of *N* = 3, **p < 0.01, ***p < 0.001 in comparison to cells incubated with vehicle for the indicated time. **(B)** AcoA, ouabain and digoxin reduce Src kinase activity in a cell-free assay. Enzymatic activity of recombinant c-Src kinase (0.1 U/mL) pretreated with AcoA, ouabain, digoxin, doxorubicin, or the positive control staurosporine (all 100 nM) for 30 min, was analyzed by a kinase assay. Data are mean ± SD of triplicates, *p < 0.05, **p < 0.01, ***p < 0.001 in comparison to the vehicle control. **(C)** Src activation (*p*-Src^Y416^ expression) in the NCI-60 tumor cell lines does not correlate with AcoA cytotoxicity (GI_50_ values) in the same cell lines (original data were obtained from NCI database). Red dot indicates A549 cell line. Pearson correlation test. **(D)** AcoA and ouabain-induced cell toxicity is unaffected by the Src inhibitor PP2. Cell viability of A549 cells pretreated for 4 h with the Src kinase family inhibitor PP2 and incubated for 48 h with AcoA or ouabain (100 nM). XTT assay, mean ± SEM of *N* = 3.

We found that AcoA inhibits the Na^+^/K^+^-ATPase in NSCLC cells, and there is evidence of a direct interaction between the Na^+^/K^+^-ATPase, Src kinase and the EGFR (Haas et al., 2002). The activation of Src kinase has been suggested as a crucial mediator of cardenolide glycoside-induced cytotoxicity in cancer cells (Diederich et al., 2017; Schneider et al., 2017). In NSCLC cells, cardenolide glycoside-induced inhibition of the Na^+^/K^+^-ATPase was shown to activate Src kinase triggering autophagic death (Wang et al., 2015). Indeed, we found that AcoA leads to an increase in Src phosphorylation in A549 cells after 1 h of exposure and lasts for several hours (Figure 4A). To further examine if AcoA directly interacts with Src kinase, we measured the activity of recombinant Src kinase in a cell-free kinase assay upon incubation with AcoA, ouabain, and digoxin. In this assay, AcoA, ouabain, and digoxin diminished the activity of recombinant Src kinase by 9.7, 18.4, and 16.6%, respectively (Figure 4B). We used the NCI-60 database to perform correlations between the available GI_50_ values of AcoA and the p-Src levels of the same cancer cell lines. There was no significant correlation between GI_50_ of AcoA and the expression levels of p-Src^Y416^ in the NCI60 cancer cell panel (Pearson’s test, *p* = 0.747, Figure 4C). Likewise, there was no significant correlation between p-Src^Y527^expression and GI_50_ values of AcoA (*p* = 0.350). Furthermore, inhibition of Src kinase by the Src family inhibitor PP2 did not affect AcoA-induced toxicity in A549 cells (Figure 4D). Thus, we concluded that AcoA-induced toxicity might not directly depend on Src kinase activation.

### AcoA-Induced Cytotoxicity Correlates to EGFR Expression in the NCI-60 Cancer Cell Panel

**FIGURE 5:**
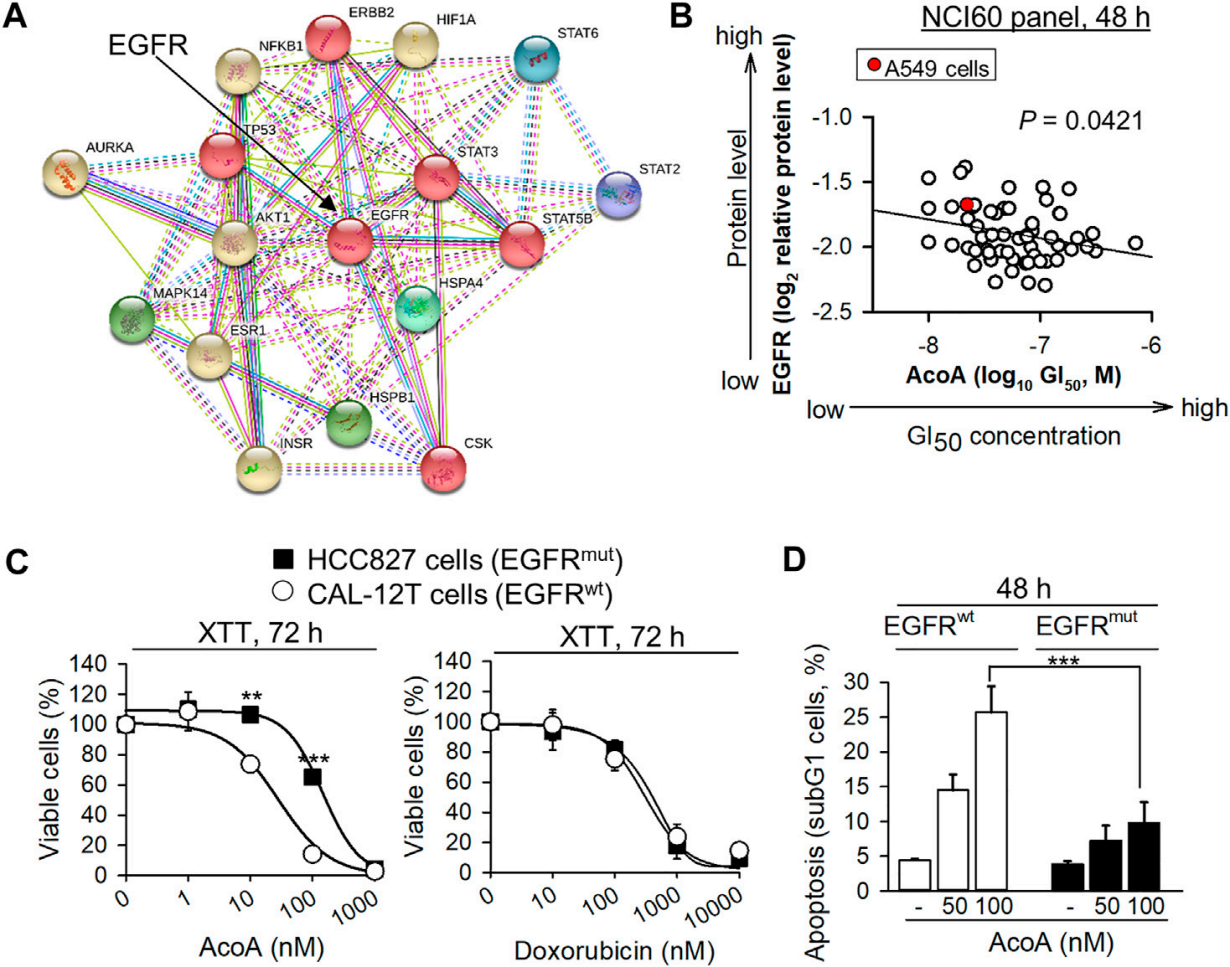
AcoA-mediated cytotoxicity in cancer cells depends on EGFR. **(A)** Functional interactions of proteins affected by AcoA according to proteome profiling arrays were analyzed by the STRING database. 6 clusters were selected. Inter-cluster edges are represented by dashed lines. **(B)** AcoA-induced GI_50_ (concentration of 50% growth inhibition) values of the NCI60 cancer cell panel positively correlate to EGFR expression levels of the respective cell lines (original data were obtained from NCI database). Red dot indicates A549 cell line. Pearson correlation test. **(C)** NSCLC cells expressing constitutively active EGFR (EGFR^mut^, HCC-827) are relatively resistant to AcoA. HCC-827 or EGFR^wt^ NSCLC cells (CAL-12T) were analyzed by XTT (*N* = 3). For comparison, the response to the conventional chemotherapeutic doxorubicin was measured in parallel (XTT, *N* = 6). **(D)** AcoA induces an apoptotic subG1 cell population in EGFR^wt^, but not in EGFR^mut^ cells as analyzed by flow cytometry of propidium iodide stained cells (*N* = 4). Data are mean ± SEM of at least *N* = 3, **p < 0.01, ***p < 0.001.

Besides Src kinase, the Na^+^/K^+^-ATPase transmembrane complex is tightly connected and interacts with EGFR (Haas et al., 2002; Diederich et al., 2017). Notably, NF-κB is a downstream target of EGFR and we found that NF-κB activity is inhibited by AcoA (Figure 1E). We performed molecular target screening using several proteome profiling arrays and found that AcoA like other cardenolide glycosides affects multiple signaling pathways in A549 cells: In particular, a phosphokinase array revealed increased phosphorylation of heat shock proteins, STAT2, STAT3 and STAT6 and AKT (Supplementary Figure S1A). Furthermore, the protein expression of HIF-1α was reduced by AcoA after 3 h and 24 h of incubation (Supplementary Figure S1B). To tackle the functional interactions between the various proteins affected by AcoA, the STRING database was employed (Szklarczyk et al., 2017) and showed that most of the affected pathways were closely linked to EGFR (Figure 5A). To validate the role of EGFR in AcoA-induced toxicity, we compared the GI_50_ (concentrations of 50% growth inhibition) data of AcoA in the NCI-60 human tumor cell lines available from the database of the United States National Cancer Institute (NCI) to EGFR log2 relative protein expression data from the same database (https://dtp.cancer.gov/discovery_development/nci-60/). By means of Pearson’s rank correlation test, we found a significant correlation between growth inhibition by AcoA and the EGFR expression levels in the NCI-60 tumor cells (Figure 5B).

### EGFR-Mutant NSCLC Cells Show Resistance to AcoA

To confirm the role of EGFR in cardenolide glycoside-induced toxicity in NSCLC cells, we compared the toxicity of cardenolide glycosides in EGFR^wt^ and EGFR^mut^ NSCLC cells. We selected HCC827, a NSCLC cell line with an activating mutation in the tyrosine kinase domain of EGFR caused by deletion of E746-A750. Whereas A549 cells express wild-type EGFR, which, upon ligand binding, is internalized and mostly degraded, delE746-E750 mutation leads to up to three-fold increased ligand-independent activation of EGFR (Lynch et al., 2004). To avoid bias due to cell-cycle differences, an EGFR^wt^ NSCLC cell line with the same proliferation kinetics as EGFR^mut^ HCC827 cells was used (CAL-12T). Indeed, HCC827 cells expressing constitutively active EGFR (EGFR^mut^) were more resistant to AcoA than CAL-12T (EGFR^wt^) cells (Figure 5C) and less susceptible to AcoA-induced apoptosis (Figure 5D), whereas the responsiveness to the conventional chemotherapeutic doxorubicin did not differ between EGFR^wt^ and EGFR^mut^ NSCLC cells (Figure 5C). Table 2 gives an overview of cardenolide glycoside IC_50_ values after 48 h in NSCLC cell lines expressing wild-type and mutant EGFR, respectively.

**TABLE 2:** IC_50_ values (nM) of cardenolide glycosides in NSCLC cell lines (48 h incubation).

	EGFR^wt^				EGFR^mut^
NSCLC cell line	A549			CAL-12T	HCC827
Cardenolide glycoside	Digoxin	Ouabain	AcoA	AcoA	AcoA
IC_50_, nM	17.4 ± 14.2	10.5 ± 20.1	22.5 ± 4.3	155.7 ± 4.4	676.6 ± 4.6

EGFR^wt^, EGFR wild-type; EGFR^mut^, EGFR mutant.

Cytotoxic effects on A549 cell viability were analyzed in XTT assays (48 h) in 3-4 independent experiments, each in quadruplicates.

### AcoA Does Not Significantly Affect EGFR Phosphorylation and Binding of EGF

**FIGURE 6:**
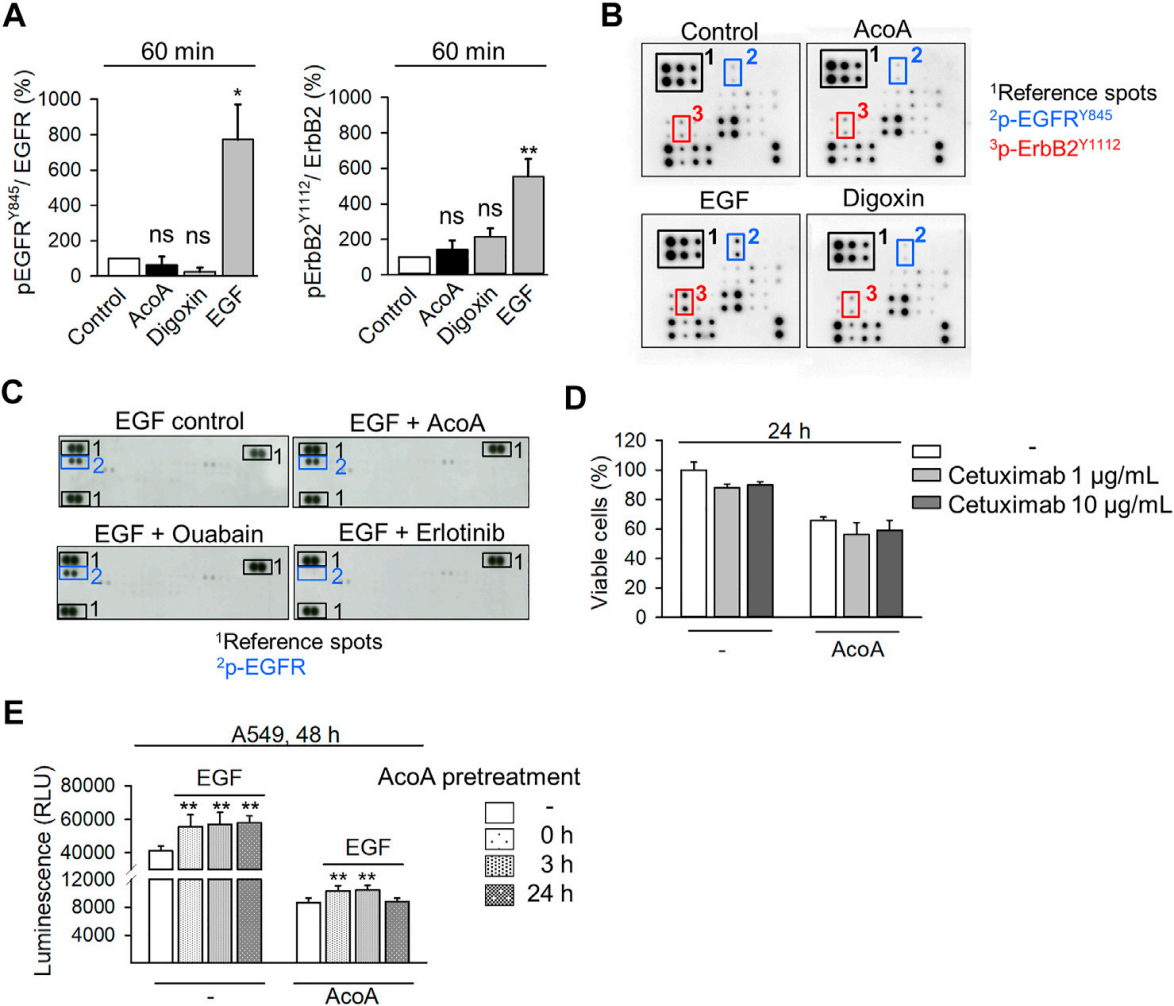
AcoA does not significantly change EGFR phosphorylation and binding and activation by EGF. **(A)** Neither AcoA nor digoxin (both 100 nM) induce phosphorylation of EGFR family members in A549 cells. EGF (100 ng/ml) was used as a positive control. Phosphorylation of EGFR^Tyr845^ and ErbB2^Tyr1112^ after 60 min treatment are quantified. Analysis was performed by protein array. **(B)** Representative membranes are shown in **(C)** Neither AcoA nor digoxin (both at 100 nM) affect EGFR phosphorylation induced by EGF (100 ng/ml) as analyzed by a tyrosine kinase protein array. A549 cells were pretreated for 30 min with AcoA, ouabain (both 100 nM), or erlotinib (10 µM) and stimulated with EGF (100 ng/ml) for 60 min. **(D)** No synergistic cytotoxicity of AcoA and the EGFR inhibitor cetuximab. A549 lung cancer cells were treated with the EGFR inhibitor cetuximab. After 1 h, AcoA (100 nM) was added and after 24 h cell viability was analyzed by XTT. Data are mean ± SEM, *N* = 3. **(E)** AcoA and digoxin do not interfere with EGF-induced cell proliferation. Cell proliferation was quantified based on luminescence in luciferase-expressing A549 cells pretreated with 100 nM of AcoA for either 0, 3, or 24 h and stimulated with 100 ng/ml EGF for additional 48 h (*N* = 3). Data are mean ± SEM of *N* = 3 independent experiments, *p < 0.05, **p < 0.01.

We examined the effects of AcoA on phosphorylation of EGFR family proteins by an antibody array, which allows the simultaneous analysis of EGFR (ErbB1), HER2/neu (ErbB2), Her3 (ErbB3), and Her4 (ErbB4) phosphorylation and includes 8 phosphorylation sites of EGFR. In this array, neither AcoA nor digoxin significantly affected any EGFR family phosphorylation site, although we observed a numerical increase of ErbB2 phosphorylation at the Tyr1112 phosphorylation site of ErbB2 (Figures 6A,B). In contrast, EGF induced phosphorylation of Tyr845 of EGFR and Tyr1112 of ErbB2 (Figure 6A). Furthermore, preincubation with AcoA did not affect the phosphorylation of EGFR by EGF, whereas erlotinib strongly suppressed EGF-induced phosphorylation (Figure 6C). Thus, the impact of cardenolide glycosides on EGFR appeared not mediated by phosphorylation.

To explore whether AcoA interferes with the ligand binding site of EGFR, we pretreated A549 cells with cetuximab (Erbitux®), a monoclonal antibody, which blocks the EGFR extracellular ligand binding site (Li et al., 2005) prior to addition of AcoA. Yet, AcoA toxicity was not attenuated by cetuximab (Figure 6D). Similarly, AcoA did not prevent EGF stimulation of cell proliferation when AcoA and EGF were added either simultaneously (0 h) or AcoA was added 3 h before EGF (Figure 6E). These results show that AcoA does not directly antagonize EGF binding and EGF-induced EGFR activation. Therefore, we assumed that the interference of AcoA with EGFR might take place intracellularly.

### AcoA Inhibits EGF-Induced EGFR Degradation and Arrests EGFR in the Endosomal Compartment

**FIGURE 7:**
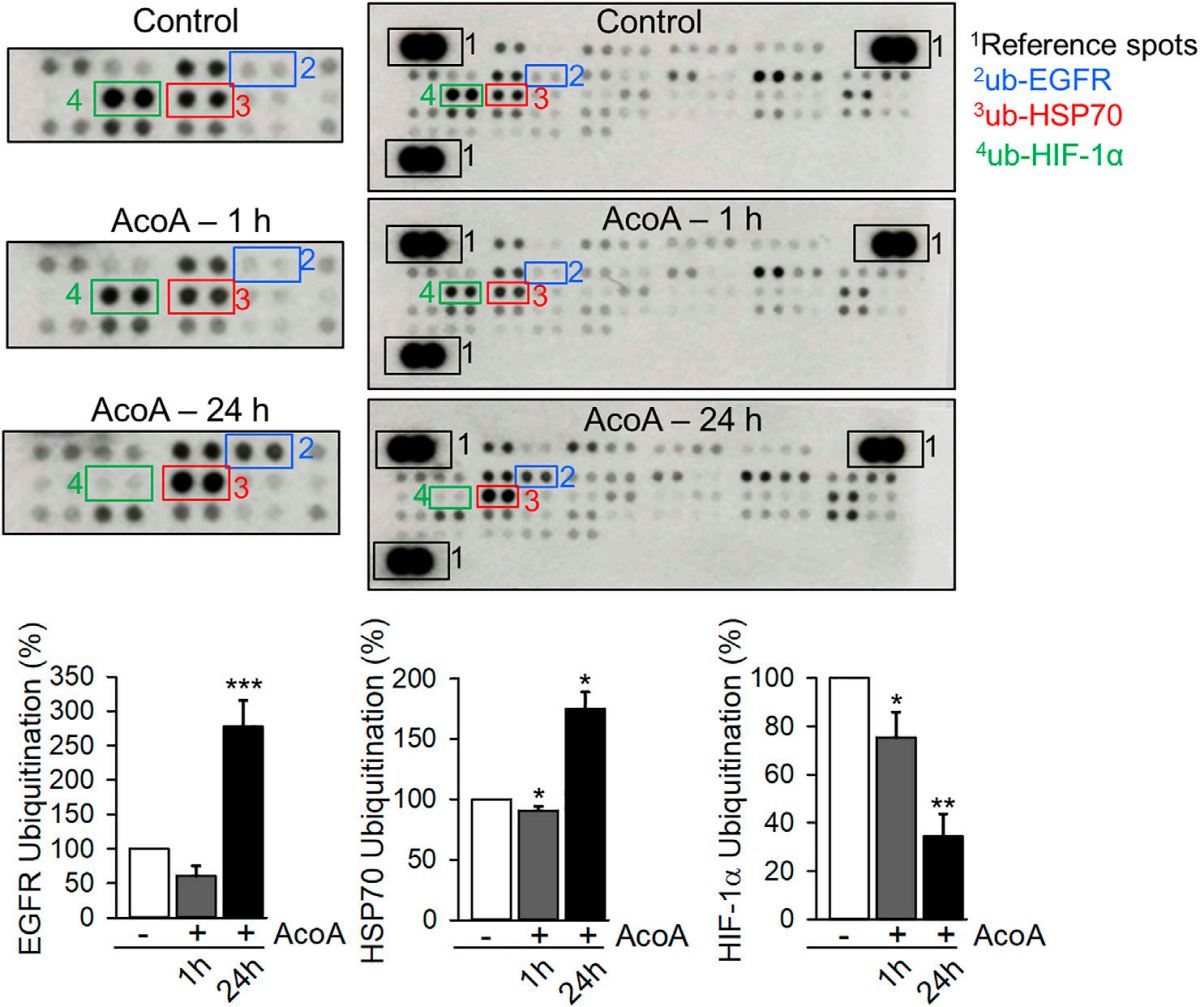
AcoA enhances EGFR ubiquitination **(A)** AcoA affects the ubiquitination of EGFR, HIF-1α and HSP70 in A549 cells. The analysis was performed by protein array. Pixel density was quantified using ImageJ software and compared to the pixel density of the control. Representative membranes are shown. Data are mean ± SEM of *N* = 3 independent experiments, *p < 0.05, **p < 0.01, ***p < 0.001 vs. vehicle-treated cells.

In addition to phosphorylation, EGFR activation and signaling is regulated by EGFR endocytosis and post-endocytic sorting (Tan et al., 2016). Ubiquitination is a necessary precondition to facilitate endosomal sorting of EGFR and ubiquitination marks activated EGFR for degradation (De et al., 2014; Tan et al., 2016). We used a proteome profiling array to analyze ubiquitination levels of EGFR along with 48 other proteins. Indeed, prolonged treatment with AcoA significantly increased ubiquitination of EGFR (Figure 7). Furthermore, we found increased ubiquitination of HSP70, a chaperone with regulatory function in stress-related protein degradation via the ubiquitin-proteasome system (Rosenzweig et al., 2019), whereas HIF-1α ubiquitination was reduced (Figure 7). Ubiquitination of other proteins was not significantly changed indicating that the observed changes in ubiquitination are rather specific.

**FIGURE 8:**
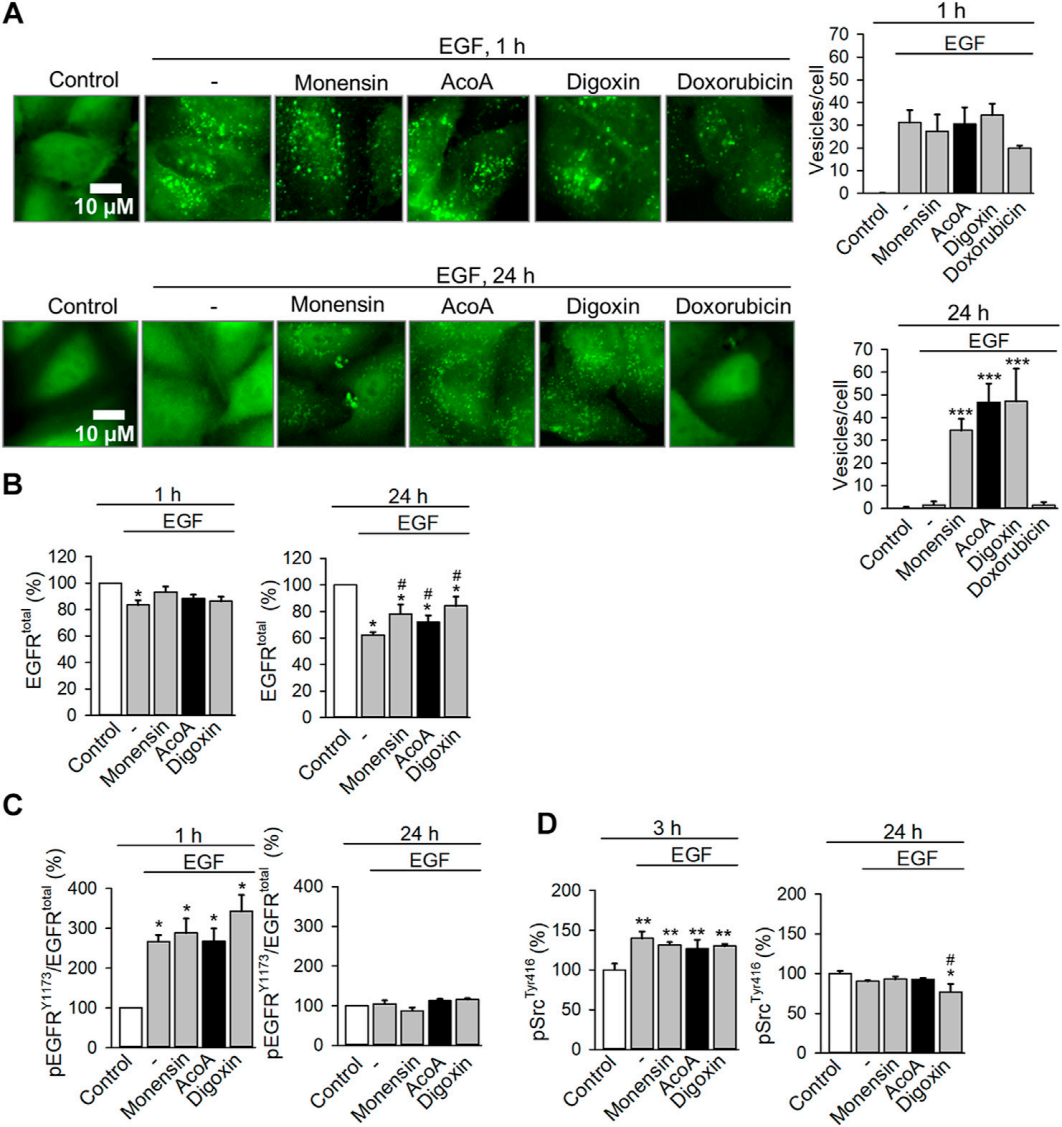
AcoA leads to endosomal EGFR arrest and inhibits EGF-induced degradation of EGFR **(A)** AcoA induces endosomal arrest in EGFR biosensor cells. After 1 h preincubation with monensin (1 µM), AcoA, digoxin, doxorubicin (all 100 nM), or vehicle, cells were stimulated with 100 ng/ml EGF and the formation of green fluorescent vesicles indicating activated and internalized EGFR was monitored microscopically. Representative images are shown. The graphs on the right show the number of fluorescent vesicles/cell at the respective time point. Data are mean ± SEM of *N* = 3 independent experiments, ***p < 0.001. **(B)** Quantification of cellular EGFR in A549 cells treated as in **(A)** using a commercial ELISA In-cell ELISA assay. The amount of EGFR was normalized to cell number assessed by crystal violet staining. Data are mean ± SEM of *N* = 3 independent experiments, *p < 0.05 vs. control, #*p* < 0.05 vs. EGF treatment group. **(C)** At the same conditions, EGFR activation was assessed using ELISA for the Y1173 phosphorylation site of EGFR. EGFR phosphorylation is expressed as the ratio of phosphorylated EGFR to total EGFR. Data are mean ± SEM of *N* = 3, *p < 0.05 vs. control. **(D)** Src kinase activation was measured as a downstream target of EGFR. A549 cells treated as in **(A)** were lysed and the cell lysates were analyzed for activation of the Tyr416 phosphorylation site of Src kinase using a commercial ELISA assay. Data are mean ± SEM of *N* = 3, *p < 0.05 vs. control, **p < 0.01 vs. control, #*p* < 0.05 vs. EGF treatment group.

Then, we analyzed EGFR trafficking using A549 EGFR biosensor cells stably expressing a fluorescent biosensor binding to activated EGFR tyrosine kinase. Activation of the receptor results in biosensor binding to EGFR with subsequent internalization via endocytosis (Malkov et al., 2011). Kinetic analyses of EGFR trafficking upon EGF stimulation showed rapid redistribution of biosensor-bound EGFR from the cell membrane to intracellular endosomes visible as fluorescent granules (Figure 8A). At first instance, this process was neither affected by AcoA nor by digoxin (Figure 8A). Under physiological conditions, most of the internalized EGFR is degraded. Accordingly, in vehicle- or doxorubicin-treated cells, EGFR-containing endosomes had disappeared after 24 h indicating EGFR degradation. In AcoA and digoxin-treated cells, however, granules with internalized receptor persisted and were not degraded (Figure 8A) indicating endosomal arrest of EGFR. The antibiotic monensin is known to inhibit the exit of internalized EGFR from sorting endosomes and prevents EGFR recycling, which was shown to induce apoptosis in cancer cells (Wang et al., 2002; Rush et al., 2012). Thus, we used monensin as a positive control and found that monensin induced endosomal EGFR arrest very similarly to AcoA and digoxin (Figure 8A).

If trafficking and degradation of internalized EGFR is blocked, this might result in an increase of intracellular EGFR. To quantify total cellular amounts of EGFR we used ELISA assays and stimulated A549 cells with EGF. We found that EGF rapidly reduced EGFR levels in A549 cells, indicating that the activated EGFR had been degraded after endosomal sorting (Figure 8B). Both, AcoA and digoxin significantly mitigated EGF-induced EGFR degradation comparable to monensin (Figure 8B).

In parallel measurements we assessed the activation state and downstream signaling of EGFR. As expected, EGF stimulation activated EGFR after 1 h (Figure 8C). In comparison to EGF, the phosphorylation of EGFR was not significantly affected by AcoA, digoxin and monensin, neither immediately nor after 24 h, although we observed a tendency to stronger activation by digoxin. To see if the EGFR downstream signaling at these conditions was affected, we checked Src activation using ELISA technique. Likewise, we did not observe changes of Src phosphorylation in comparison to EGF after short incubation time. Only after prolonged exposure for 24 h we observed a numerical and for digoxin also a significant decrease of Src kinase activation. Thus, it is unlikely that the observed EGFR arrest is driven by changes in EGF-induced receptor activation and Src kinase signaling.

Monensin is also a known Na^+^ ionophore as it transports Na^+^ ions across the cell membrane (Huczynski et al., 2012), and Na^+^ concentration modulates cellular signaling and EGFR trafficking (Lee et al., 2015). We wondered if the observed effects of both, cardenolide glycosides and monensin, might be related to their potency to increase intracellular Na^+^. NF-κB activation is reduced by cardenolide glycosides (Figure 1E), and NF-κB activation was shown to depend on Na^+^ concentration (Vinciguerra et al., 2005). Accordingly, we hypothesized that the Na^+^ ionophore monensin might affect NF-κB activation as well. Using the same NF-κB reporter cell line and conditions as in Figure 1E, we found that monensin concentration-dependently inhibited TNF-α-induced activation of NF-κB (Supplementary Figure S4).

## Discussion

**FIGURE 9:**
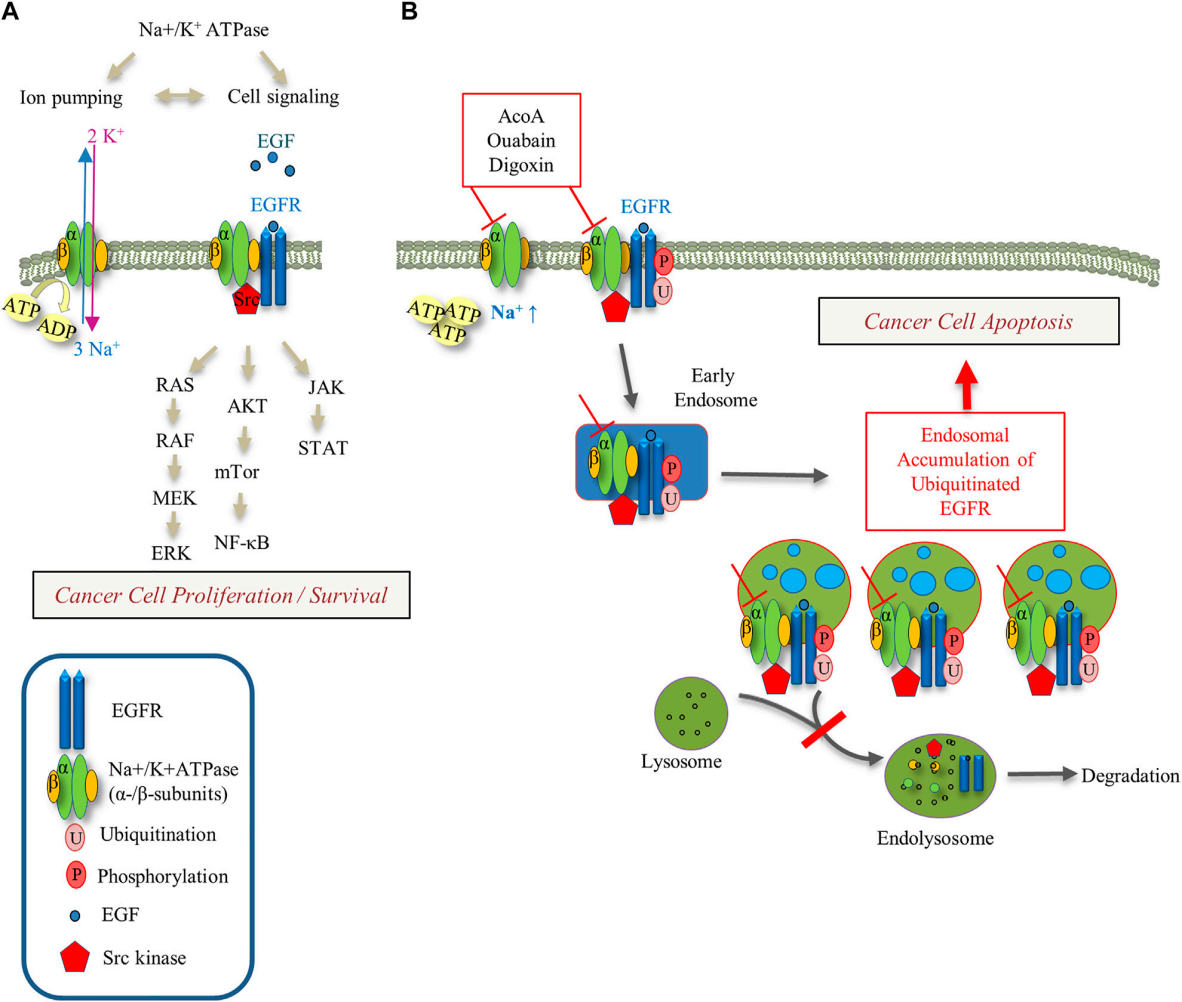
Schematic of Na^+^-K^+^-ATPase and EGFR signaling pathways and their modulation by cardenolide glycosides. **(A)** The interaction between the Na^+^/K^+^-ATPase, EGFR and Src kinase regulates downstream signaling pathways with impact on cancer cell proliferation and survival. **(B)** Cardenolide glycoside-induced inhibition of the Na^+^/K^+^-ATPase ion pumping function increases intracellular Na^+^ and ATP levels. Inhibition of the Na^+^/K^+^-ATPase increases intracellular Na^+^ and inhibits EGF-induced EGFR degradation yielding endosomal EGFR arrest. This perturbation of EGFR trafficking may trigger apoptotic cell death.

Our study shows that AcoA shares typical pharmacodynamic features of cardenolide glycosides, and leads into apoptotic cell death of A549 NSCLC lung cancer cells. Our data further indicate interference of AcoA with EGFR trafficking and EGFR downstream signaling. We discuss our findings with a focus on the relationship between the EGFR and intracellular Na^+^ concentrations (See Figure 9).

### Increase of Na^+^ and ATP as Indirect Measures of Na^+^/K^+^-ATPase Inhibition

We show that AcoA increases intracellular Na^+^ and ATP concentrations (Figures 1B–D). In A549 cells, the Na^+^/K^+^-ATPase accounts for up to 75% of total cellular ATP turnover (Bogdanova et al., 2016). Therefore, increased intracellular ATP levels upon cardenolide glycoside treatment indicate that the ion transport has been blocked and the Na^+^/K^+^-ATPase is efficiently inhibited (Figures 1D, 2D).

### Interaction Between Cardenolide Glycosides, Src Kinase and the EGFR

The activation of distinct kinases, including Src kinase, has been described as a typical feature of cardenolide glycosides (Prassas et al., 2011; Diederich et al., 2017) and was previously made responsible for digoxin-mediated cancer cell death (Wang et al., 2015). Indeed, we also found that AcoA activates Src kinase in A549 cells (Figure 4A). However, this did not apply in a cell-free assay using recombinant Src kinase and externally added ATP (Figure 4B) and in digoxin-treated A549 cells Src kinase activity was reduced after 24 h (Figure 8D). Furthermore, the analysis of AcoA-induced growth inhibition in the NCI-60 tumor cell line panel did not show significant correlations with the expression of *p*-Src, and pre-treatment with the Src kinase inhibitor PP2 did not affect AcoA-induced cytotoxicity (Figures 4C,D). Therefore, our data do not support the hypothesis that Src kinase activation directly mediates cardenolide glycoside-induced NSCLC cell toxicity. However, Src kinase relies on endosomes for the delivery from the perinuclear region to the plasma membrane to become activated and it has been shown that Src family kinases and receptor tyrosine kinases utilize the same endosomal pathways (Sandilands and Frame, 2008). We show that cardenolide glycosides affect endosomal trafficking of the receptor tyrosine kinase EGFR. It can be speculated that endosomal trafficking of Src kinase is dysregulated by cardenolide glycosides as well.

### EGFR Trafficking and Implication of Intracellular Na^+^ Concentrations

Src kinase is a downstream target of the EGFR and based on a NCI-60 database query EGFR emerged as a possible determinant of cardenolide glycoside-dependent toxicity (Figure 5B). Indeed, we found that AcoA and digoxin affect EGFR trafficking and signaling. EGF stimulation leads into EGFR internalization and endosomal sorting, and ubiquitination marks the EGFR for further degradation. Our data indicate that cardenolide glycosides do not hamper EGFR activation and internalization, whereas accumulation of the ubiquitinated EGFR upon exposure to AcoA indicates that EGFR after endosomal sorting is not further processed (Figure 6).

To assess ubiquitination we used a screening array comprising 49 different proteins including other tyrosine kinase receptors. Apart from EGFR, only HSP70 and HIF-1α ubiquitination were affected by AcoA (Figure 7), which indicates specificity of this effect. HSP70 plays a regulatory role in protein degradation and the EGFR is one of its substrates (Fernandez-Fernandez et al., 2017). In tumor cells, HSP70 is located within the membranes of endo-lysosomes (Nylandsted et al., 2004). Thus, accumulation of HSP70 corroborates the hypothesis that ubiquitinated EGFR is indeed arrested in the endo-lysosomal compartment.

Quantification by ELISA and microscopic visualization confirmed that the EGF-induced degradation of EGFR is significantly decelerated by AcoA (Figure 8). This was comparable to monensin, which served as a positive control. Monensin is a Na^+^ ionophore and has been shown to slow down EGF-induced EGFR degradation in cancer cells (Rush et al., 2012). Rush et al. used immunoblots and monitored EGFR levels in HeLa cells for 3 h. Our findings using ELISA technique and A549 cells confirm these data, and we additionally show that the inhibition of EGFR degradation by monensin and cardenolide glycosides persists after continued exposure for at least 24 h (Figures 8A,B). Interestingly, EGF itself has been shown to enhance Na^+^ influx upon binding to EGFR (Rothenberg et al., 1983) and Na^+^ influx by EGF induces microtubule acetylation which is essential for EGFR trafficking along microtubules (Deribe et al., 2009; Lee et al., 2015). Like EGF, monensin as a Na^+^ ionophore, and cardenolide glycosides as inhibitors of the Na^+^/K^+^-ATPase enhanced microtubule acetylation via deactivation of histone deacetylase 6 and accumulation of acetylated tubulin (Lee et al., 2015). Lee et al. further showed that the speed of EGF-induced EGFR trafficking is associated to intracellular Na^+^ concentrations with higher Na^+^ slowing down EGFR trafficking (Lee et al., 2015). This is in line with our findings of endosomal EGFR arrest and reduced EGFR degradation upon application of monensin and the cardenolide glycosides AcoA and digoxin (Figures 8A,B).

### NF-κB Activation in Light of Intracellular Na^+^ Concentrations

Sodium homeostasis is restored and controlled by cellular Na^+^/K^+^-ATPase, and therefore efficient EGFR trafficking relies on the Na^+^/K^+^-ATPase ion pumping function. EGFR signaling activates NF-κB via the IKK complex and IκBα (Shostak and Chariot, 2015), whereas inhibition of NF-κB is a common feature of several members of the cardenolide glycoside family related to cancer cell mitotic arrest and apoptosis (39–41). In A549 cells, NF-κB signaling is highly activated and cardenolide glycosides have been shown to reduce both, DNA binding and transcriptional activity of NF-κB (Mijatovic et al., 2006). We observed previously that AcoA inhibits constitutively active NF-κB (Ezzat et al., 2016). Now, we show that AcoA strongly inhibits TNF-α-induced NF-κB activity as well (Figure 1E). Interestingly, NF-κB inducers like TNF-α enhance Na^+^ transport and induce cell-surface Na^+^/K^+^-ATPase recruitment in kidney cells (Vinciguerra et al., 2005). With increasing Na^+^ concentrations, protein kinase A dissociates from the catalytic subunit of p65- NF-κB and IκBα, which reduces TNF-α induced NF-κB activation (Vinciguerra et al., 2005). Thus, inhibition of the Na^+^/K^+^-ATPase by cardenolide glycosides might counteract TNF-α induced NF-κB activity via the elevation of Na^+^ concentrations. The association between NF-κB activation and Na^+^ concentration is further corroborated by our finding that the Na^+^ ionophore monensin inhibits NF-κB activation as well (Supplementary Figure S4).

### Susceptibility of Cancer Cells to Changes in EGFR Trafficking and Na^+^ Homeostasis

For cancer cell survival and proliferation, maintenance of Na^+^ homeostasis is essential, and synthetic ion transporters increasing intracellular Na^+^ concentrations were shown to induce osmotic stress, generate reactive oxygen species, and induce caspase-dependent cell death in A549 NSCLC and other cancer cells (Park et al., 2019). EGFR trafficking is regulated by intracellular Na^+^ concentrations (Lee et al., 2015). Thus, EGFR-expressing cancer cells might be more vulnerable by altered Na^+^ concentrations than non-tumor cells with lower EGFR expression levels.

Rush et al. showed that inhibition of EGFR degradation by monensin leads into accumulation of EGFR on the endosomal membrane of MDA-MB-468 breast cancer cells and co-treatment with EGF induces apoptosis in HeLa cells (Rush et al., 2012). The apoptotic cell death upon EGF addition might be due to the synergistic effect on Na^+^ concentrations and EGFR trafficking by both, EGF and monensin. In our experiments in A549 cells, AcoA did not prevent EGF-stimulated cell proliferation (Figure 6E). However, as the effect observed by Rush et al. in HeLa cells was very small, we assume that it depends on the specific experimental conditions and the characteristics of the cell line: HeLa cells express EGFR at much higher density and with different binding kinetic constants than A549 cells (Zhang et al., 2015). Therefore, the increase in intracellular Na^+^ induced by EGF might be more pronounced in HeLa cells than in A549 cells. Furthermore, Rush et al. applied serum starvation conditions, and serum starvation has been shown to induce endosomal EGFR arrest itself (Tan et al., 2016). In addition, ionic transporters and channels might be differentially expressed in HeLa vs. A549 cells as well, with influence on the capacity to balance out slight changes of ionic gradients induced by EGF.

Our findings of AcoA-induced EGFR arrest are not sufficient to prove that this triggers A549 cell death. However, the association between AcoA-induced cell death and EGFR trafficking is corroborated by the differential susceptibility of EGFR-mutant NSCLC cells: We found that EGFR-mutant HCC827 with constitutively active EGFR are resistant to AcoA (Figures 5C,D, Table 2). Chung et al. showed that in mutant EGFR the endocytic recycling pathway plays a major role, whereas in EGFR wild-type cells, EGFR is mostly degraded (Chung et al., 2009). Thus, HCC827 NSCLC cells with constitutively active EGFR might be less dependent on successful EGFR degradation than EGFR wild-type cells.

Further studies may substantiate the impact of our findings in EGFR wild-type in comparison to EGFR-mutant NSCLC cells in the light of Na^+^ levels and EGFR trafficking. Furthermore, the interactions between the Na^+^/K^+^-ATPase, intracellular Na^+^ concentrations, Src kinase and EGFR trafficking and signaling merit further exploration. Another important point is whether these interactions apply *in vivo* and might be utilized to improve lung cancer treatment.

### Clinical Application of Cardenolide Glycosides – Opportunities and Limitations

Considering the clinical application of cardenolide glycosides, therapeutic plasma concentrations of digoxin are in the range of 1 nM to 2.6 nM, whereas digoxin is very likely to display symptoms of toxicity above 3.8 nM (Goldberger and Goldberger, 2012). Thus, non-toxic concentrations of digoxin in humans are lower than the concentrations needed to affect Na^+^/K^+^-ATPase-mediated EGFR trafficking in our experiments. However, a considerable part of the anti-tumor efficacy of cardiac glycosides *in vivo* might be due to interaction with the tumor environment. Previously, digitoxin was shown to inhibit angiogenesis at therapeutic concentrations *in vitro* in human endothelial cells and *in vivo* in ovarian cancer xenografts (Trenti et al., 2017). We observed reduced vascularization in tumor xenografts topically treated with AcoA or ouabain (Supplementary Figure S3). Likewise, a Swedish group found that digoxin inhibits FGF-2-stimulated angiogenesis in the CAM assay and suggested digoxin as an unspecific inhibitor of angiogenesis (Svensson et al., 2005).

Regarding clinical applicability and safety, AcoA might display several advantages over other cardiac glycosides: In toxicological studies performed in rodents, AcoA showed much lower systemic toxicity than all cardenolide glycosides currently used in patients. Thus, upon intraperitoneal administration in mice, the LD_50_ of AcoA was 223 mg/kg (Ezzat et al., 2016), which implies more than 50-fold lower toxicity than digoxin (LD_50_ 4 mg/kg (Frayha et al., 1971)) and 20-fold lower toxicity than ouabain (LD_50_ 11 mg/kg (Forster et al., 1965)). When these results are translated to humans, AcoA could be applied at much higher dosages compared to digoxin and may reach efficient concentrations to combat cancer cells without the crux of inducing systemic toxicity. Predicted pharmacokinetics of AcoA suggest that AcoA is well absorbed upon gastrointestinal administration making it suitable for oral administration in patients (see Table 1). Ouabain contains many hydroxyl groups resulting in poor gastrointestinal absorption due to high polarity (Figure 1A). Digoxin is poorly absorbed upon oral intake as well, maybe due to three sugar moieties and relatively high molecular weight impeding mucosal resorption (see Table 1 and Figure 1A). Thus, AcoA exhibits more favorable properties for clinical use than digoxin and ouabain.

## Conclusion

In conclusion, our data confirm that AcoA displays typical pharmacodynamic features of the cardenolide glycoside family and exhibits cytotoxic potential in NSCLC cells equivalent to digoxin and ouabain. We show that AcoA affects EGFR-dependent signaling via Src kinase and dysregulates EGFR trafficking similar to the Na^+^ ionophore monensin. We suggest that the observed increase of intracellular Na^+^ concentration likely is related to aberrant EGFR trafficking. Thereby, we propose a novel mechanism of cardenolide glycoside-induced impairment of EGFR-expressing cancer cells and intend to encourage the further pharmacological exploration of these naturally-derived compounds.

## Data Availability

The original contributions presented in the study are included in the article/Supplementary Material, further inquiries can be directed to the corresponding author.
