# Supplementary material for: The Cardenolide Glycoside Acovenoside A Interferes with Epidermal Growth Factor Receptor Trafficking in Non-Small Cell Lung Cancer Cells
Source: Front Pharmacol. 2021 May 5;12:611657. doi: 10.3389/fphar.2021.611657 (PMC8133365; doi:10.3389/fphar.2021.611657)
Supplement: Supplementary file 1 [file DataSheet1.DOCX]

Supplementary Material

# Supplementary Materials and Methods

## Cell lines

Mouse J774.1 reporter cells (InvivoGen, San Diego, CA) are macrophage-like cells used for the study of the NF-kB pathway by the activity of a secreted embryonic alkaline phosphatase (SEAP). Cells were cultured in DMEM medium supplemented with 2 mM L-glutamine, 4.5 g/L glucose, 3.7 g/L sodium bicarbonate, 1.0 mM sodium pyruvate, and 100 µg/mL normocin.

# Supplementary Figures

**
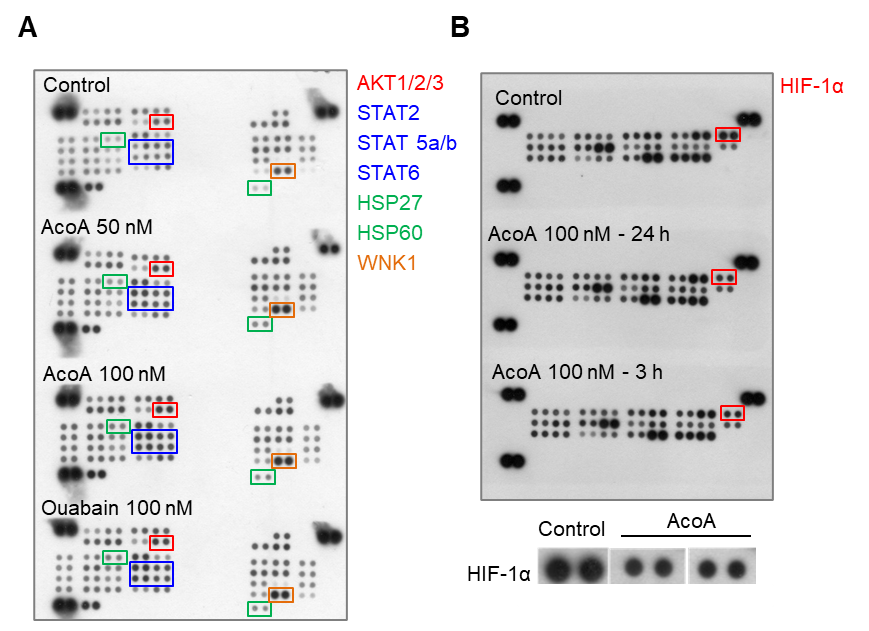
**

**Supplementary Figure S1.**

Screening for molecular targets by Proteome Profiler Arrays reveals that AcoA affects the phosphorylation level of multiple kinases and reduces the expression of HIF-1α in A549 lung carcinoma cells (**A**) Membrane of Human Phosphokinase Array to measure the relative level of human kinase phosphorylation after 90 min incubation with 0.5% DMSO (control), AcoA or ouabain. (**B**) Membrane of Human Cell Stress Array to measure the relative expression levels of stress-related proteins, incubation with 0.5% DMSO (control) or AcoA.


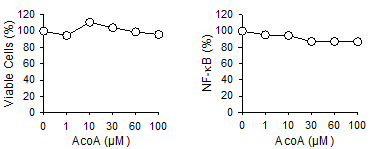


**Supplementary Figure S2.**

J774.1 mouse macrophage cells exhibit AcoA resistance. Mouse J774 NF-κB reporter cells were used to analyze mouse cell sensitivity to AcoA. Cells were treated with AcoA and after 24 h, supernatants were collected and analyzed. Cell viability was measured by XTT assay, NF-κB activation was analyzed by SEAP reporter protein expression. Data are mean ± SEM, n = 3.


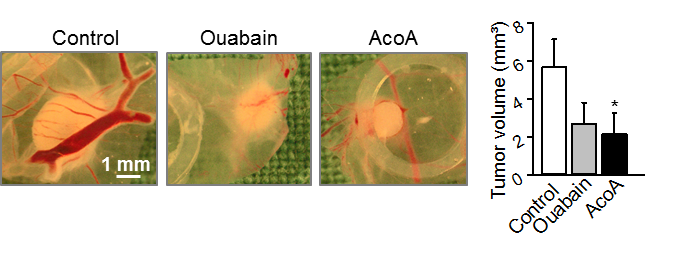


**Supplementary Figure S3.**

AcoA inhibits growth of lung cancer xenografts on the chorioallantoic membrane of fertilized chick eggs *in vivo*. 1x10^6^ A459 cells were xenotransplanted onto the chorioallantoic membrane of fertilized chick eggs 7 days after fertilization. Starting the next day, the tumors were treated with 100 nM AcoA, ouabain, or solvent (0.5% DMSO) for 3 days. Tumor volume (mm³) was calculated by length x width² x π/6. The figure shows representative pictures of tumor xenografts immediately after extraction and mean tumor volumes. Data are mean ± SEM of 5-10 eggs/group, **P* < 0.05.

**
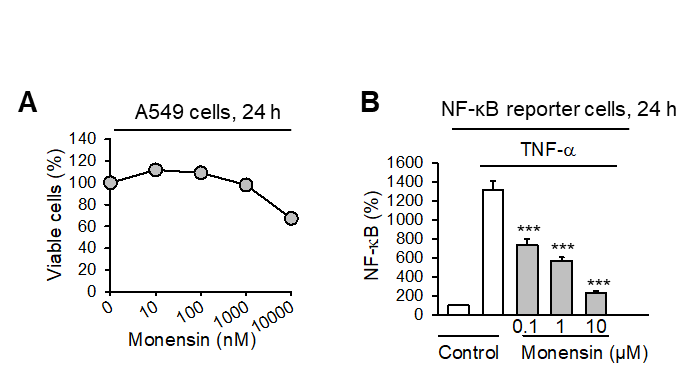
**

**Supplementary Figure S4.**

Monensin inhibits TNF-α-induced NF-κB activation at non-toxic concentrations. **(A)** Cell viability of A459 cells treated with monensin for 24 h, XTT assay, mean ± SEM, n = 4. **(B)** NF-κB reporter cells seeded in 96-well plates were treated with monensin and stimulated with TNF-α (100 ng/mL) for 24 h. Supernatants were collected and the activation of NF-κB was analyzed by SEAP reporter protein expression. Data are mean ± SEM, n = 3., ****P* < 0.001 in comparison to vehicle-treated cells stimulated with TNF-α.
